# Editorial: Shared decision making in pediatric differences/disorders of sex development

**DOI:** 10.3389/fruro.2023.1281181

**Published:** 2023-09-08

**Authors:** Kristina Suorsa-Johnson, Rebecca K. Delaney, Angela Fagerlin, David E. Sandberg

**Affiliations:** ^1^ Division of Psychiatry and Behavioral Health, Department of Pediatrics, University of Utah Spencer Fox Eccles School of Medicine, Salt Lake City, UT, United States; ^2^ Department of Population Health Sciences, University of Utah Spencer Fox Eccles School of Medicine, Salt Lake City, UT, United States; ^3^ Veterans Administration Health Services Research and Development Informatics, Decision-Enhancement and Analytic Sciences Center, Veterans Administration Salt Lake City Health Care System, Salt Lake City, UT, United States; ^4^ Susan B. Meister Child Health Evaluation & Research (CHEAR) Center, University of Michigan, Ann Arbor, MI, United States; ^5^ Division of Pediatric Psychology, Department of Pediatrics, University of Michigan Medical School, Ann Arbor, MI, United States

**Keywords:** shared decision making, decision making, differences of sex development, disorders of sex development, intersex, education

Differences/disorders of sex development **(**DSD**)** is an umbrella term for a wide variety of congenital conditions in which the development of chromosomal, gonadal, or anatomic sex is atypical (1). DSD are associated with increased incidence of infertility (2–4), gonadal cancer risks (5–8), hypogonadism (9, 10), and with anatomic and cosmetic differences perceived as obstacles to satisfying sexual function (11–14). Individuals with DSD also experience an elevated risk of gender dysphoria (15–18), psychosocial distress (19–21), and pervasive challenges to psychosocial adaptation (22–26). Although heterogeneity in presentation exists (e.g., genotype, phenotype), salient commonalities exist across DSD, including the need to make decisions about care when there is no one “best” answer for all patients and families.

Decision making in pediatric DSD is further complicated by: 1) controversial interventions (e.g., gonadal/genital surgery) that are rarely urgent; 2) decisions are commonly made when the child is too young to be meaningfully involved in the process; 3) there is substantial uncertainty regarding the potential harms and benefits associated with each option – whether performing or withholding; and 4) healthcare providers may have differing opinions about what constitutes appropriate medical care. Also, these decisions are recognized to be highly value sensitive and the available evidence regarding the short- and long-term benefits and risks of options may be limited, unclear, or subject to debate.

Parents are often unaware there are any decisions to be made; instead believing that necessary treatments follow directly from diagnosis (27, 28). Some perceive clinicians as biasing parents’ healthcare decisions on behalf of their child, rather than sharing information about all reasonable options (29–33). Evidence suggests parents receive ambivalent messages about DSD care, for example: while clinicians express caution, they also present genital surgery as beneficial without sharing adequate information about potential harms and alternative options (34). More generally, parents of infants and young children with a DSD may not be meaningfully involved in decision making (35).

Shared decision making (SDM) has been referred to as the “crux of patient-centered care” (36). A healthcare provider implementing SDM: 1) clearly informs their patient that there is a decision to be made; 2) provides the best evidence available on all decision options, including associated benefits and harms; and 3) supports the patient to identify their preferred option by discussing the patient’s preferences for each option outcome (37). When patients are unable to meaningfully participate in decision making because of young age, SDM may occur between providers and parents. Despite the benefits, barriers to SDM implementation persist; this includes both practice (time, poor fit with workflow, and lack of information designed for patient use) and systemic issues (attitudes of the provider/patient-parent and that SDM is not a standard part of care) (38, 39). SDM is broadly endorsed by national and international medical societies and policy makers, yet there is strong evidence of barriers and resistance to its implementation (40, 41). This Research Topic was conducted to add to the literature by providing information about elements of SDM, SDM approaches, and barriers to implementation in pediatric DSD.

In the first article in this Research Topic, Gardner et al. focus on understanding the decision support experiences and outcomes faced by parents of a child with DSD. The most difficult decisions identified related to surgery and disclosure of the child’s condition to others. Many parents worried about making or having made the “wrong” decision. Parents preferred being involved in decision-making, but experienced emotional distress and informational concerns that underscore the need for formalized decisional support.

To understand the nature of surgical decision making in DSD, Weidler et al. conducted qualitative interviews with key stakeholders (adults and teenagers with DSD, parents of children with a DSD, DSD-specialized healthcare providers, and allied professionals). Participants indicated different reasons for pursuing surgery and parental worries over decisional regret was noted as an ongoing challenge. There was consensus regarding patient involvement in decisions when “developmentally appropriate.” Overall, results reinforce the need for increased educational and decision support to facilitate SDM in DSD surgical decisions.

Lightfoot et al. described the development and alpha testing of a suite of four parent-proxy decision aids for DSD: genetic testing, gender of rearing, genital surgery, and gonadal surgery. Development was guided by the Ottawa Decision Support Framework (42) and the International Patient Decision Aids Standards (43).

The mixed methods study by Lapham et al. examined the opinions of North American pediatric urologists and endocrinologists regarding informational elements of informed consent. A majority of participants from both specialties agreed or strongly agreed with adding four statements to informed consent documents for genital surgery in DSD that far exceed the detail commonly seen in such documents.

Finally, Roen et al. engaged healthcare professionals, parents, and young people with DSD in interviews to learn about provider and parent education of the youth about their medical condition. The provocative title “Whose responsibility is it to talk with children and young people …” identifies an important weakness in the healthcare of these patients: who and how effective are those individuals – parents and/or providers – in imparting information to affected youth in a manner that is developmentally meaningful and material to any future decision making. The authors call for a “default plan” to prevent this critical topic from being neglected.

Overall, this Research Topic illustrates a growing trend in DSD toward studies providing balance to the biomedical aspects of clinical management with attention to the decision-making needs and values of individual patients and families in front of us.

## Author contributions

KS-J: Conceptualization, Writing – original draft, Writing – review & editing. RD: Writing – review & editing. AF: Writing – review & editing. DS: Conceptualization, Writing – original draft, Writing – review & editing.
